# Development of a Multifunctional Edible Coating and Its Preservation Effect on Sturgeon (*Acipenser baeri♀* *× Acipenser schrenckii♂*) Fillets during Refrigerated Storage at 4 °C

**DOI:** 10.3390/foods11213380

**Published:** 2022-10-27

**Authors:** Chunming Tan, De Pang, Ruiyun Wu, Fanglei Zou, Bo Zhang, Nan Shang, Pinglan Li

**Affiliations:** 1 Beijing Laboratory for Food Quality and Safety, College of Food Science and Nutritional Engineering, China Agricultural University, Beijing 100083, China; 2 Key Laboratory of Precision Nutrition and Food Quality, Department of Nutrition and Health, China Agricultural University, Beijing 100083, China; 3 College of Engineering, China Agricultural University, Beijing 100083, China

**Keywords:** sodium alginate, edible coating, sturgeon fillet, freshness preservation, refrigeration

## Abstract

Although many coatings and films can improve the quality and shelf life of fish fillets during refrigerated storage, a more multifunctional coating material is needed. In this study, an edible alginate/protein-based coating solution was prepared by incorporating antimicrobial agents. The coating properties were characterized and its effects on the quality and shelf life of sturgeon fillets during refrigeration (4 °C) were investigated. Compared with sodium alginate coating (2% sodium alginate + antibacterial agents, H), the composite coatings (2% sodium alginate + antibacterial agents + 1:15 or 1:10 protein solution, HP-15 and HP-10) exhibited a more stable structure and better light, gas, and water barrier properties, and showed better quality-preservation effects on sturgeon fillets. The composite coatings treatments, especially HP-10 composite coating, exhibited significant (*p* < 0.05) effects in inhibiting microbial growth, maintaining sensory quality, reducing the production of total volatile basic nitrogen (TVB-N), decreasing nucleotide breakdown, and delaying the lipid oxidation and protein degradation in fillets. These findings confirm that the composite coatings can be used as a multifunctional coating material for freshness preservation of sturgeon fillets to improve quality and extend shelf life.

## 1. Introduction

Sturgeon is one of the most economically important and precious freshwater fish, and it was categorized as the second class protected in the Convention on International Trade in Endangered Species of Wild Fauna and Flora (CITES) [1]. In China, sturgeon aquaculture has developed rapidly in recent years, where the production of sturgeon accounts for 85% of the total production in the world [2]. Sturgeon is popular among consumers for its palatability, high nutritional value, and deliciousness. In food processing, sturgeon is mainly used for caviar and minced fish products such as fish balls, fish cake, and sausages [1]. Sturgeon meat accounts for 40% of the fish weight, but it is not fully utilized since sturgeon is used mainly for caviar. Its meat is beneficial to human health due to its high protein content and enriched eight essential amino acids and polyunsaturated fatty acids (PUFAs) (about 35%) [3]. At present, the sales of refrigerated sturgeon fillets continue to grow due to the changing consumption customs of the younger generation. However, the fillet is prone to deterioration because of high water activity, neutral pH, and abundant nutrition. Microbial growth and biochemical reactions in fillets lead to rapid accumulation of undesirable metabolites such as biogenic amine, total volatile basic nitrogen, and hypoxanthine, ultimately causing economic losses and endangering human health [4]. Furthermore, aquatic products are prone to oxidation due to the presence of large amounts of PUFAs, which can negatively impact color, protein integrity, and flavor [5]. Therefore, study on improving quality and extending the shelf life of fresh fillets more effectively during refrigerated storage is potentially useful, which will benefit the aquatic industry and consumers. 

Previous studies have indicated that there are some factors known to accelerate food degradation such as temperature, dehydration, and microbial growth [6,7,8], especially the microbial metabolic activities, which cause the fish spoilage by affecting the pH, the degradation of nutrients, the generation of TVB-N, the production of thiobarbituric acid reactive substances (TBARS), etc. [9]. The methods to maintain quality and delay the microbial spoilage process of aquatic products are to change the preservation conditions (chilled or frozen) and use some quality control techniques (e.g., storage temperature, packaging atmosphere, preservatives, etc.) [10,11,12]. Generally, aquatic industries use the cold chains of freezing technique and cooling technique to ensure food transportation and storage under low-temperature conditions. However, some microorganisms (such as *Aeromonas*, *Pseudomonas,* and *Lactobacillus*) can still multiply at low temperatures below 5 °C [13]. Temperature control does not prevent foods from spoiling during storage, it only decreases the rate of deterioration [14,15]. Although many quality control techniques (e.g., numerous chemical additives, vacuum packaging, other preservatives, etc.) have been successfully used in the preservation of fishery products, their use is becoming limited due to the effects, cost, and health concerns they may bring about [8]. 

Natural preservatives such as ε-polylysine (ε-PL), chitosan (CS), and tea polyphenols (TP) with excellent antioxidant and antimicrobial properties have been used as safe alternatives in fish and crustaceans processing [8,11,16]. Among them, the CS is the second most abundant polysaccharide on Earth after cellulose, with antimicrobial activity, biocompatibility, and film-forming properties, and numerous researchers have investigated the effects of chitosan-based coatings on fish preservation [17]. While the polyphenols have strong antibacterial properties and effective antioxidant capacity [16,18], treatment with only one natural preservative has limited inhibitory effect on microorganisms, and some have negative effects on food organoleptic properties. In order to effectively prevent microbial spoilage and maintain the quality and safety of fillets during storage, the combination of different antimicrobial agents into edible films and coatings is necessary. In addition, bio-based films and coatings are considered to be an effective and environmentally friendly method for extending the shelf life of foods and have been extensively studied [19]. Noshad [20] and Behbahani et al. [21] demonstrated that edible coating incorporated with essential oil can effectively inhibit the growth of microbiota and reduce the lipid oxidation in meat. Yu et al. [4] indicated that chitosan coating combined with glycerol monolaurate maintained sensory quality, reduced the formation of volatile bases and oxidation products, and prolonged the shelf life of refrigerated grass carp (*Ctenopharyngodon idellus*) fillets. However, none of the developed materials exhibited multiple properties in preservation effect, material flexibility, edibility, washability, and appearance, suggesting the need for a more multifunctional coating material. In this study, we explored an edible coating based on egg protein and alginate, which combined different antimicrobial agents (including chitosan, ε-polylysine, and tea polyphenol). We aimed to study the effects of this edible coating on the quality and shelf life of sturgeon fillets during refrigerated storage. 

## 2. Materials and Methods

### 2.1. Materials

The bacterial strain used in this study was *Aeromonas sobria* LT-101 (As, GenBank accession number OK103778), which was a specific spoilage organism of refrigerated sturgeon fillets. Egg white and egg yolk powder, ε-polylysine, and tea polyphenol were obtained from Shanghai Yuanye Bio-Technology Co., Ltd. (Shanghai, China). Food-grade alginate was obtained from Shanghai Macklin Biochemical Co., Ltd. (Shanghai, China). Chitosan was obtained from Jinan Haidebei Marine Bioengineering Co., Ltd. (Jinan, Shandong, China), at the molecular weight of 300–400 KDa and deacetylation of 90%. Analytical-grade sodium hydroxide pellets and glycerol were obtained from Sigma-Aldrich (Shanghai, China). 

### 2.2. Preparation of Coating Solution 

**Figure 1:**
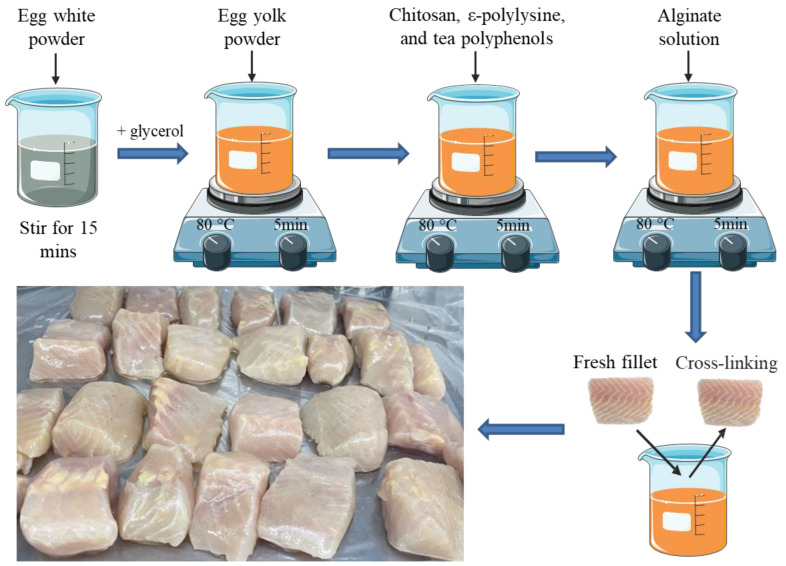
The schematic diagram of the experimental design.

We added 2% (*w/v*) sodium alginate into deionized water and stirred at 80 °C until clear to prepare an alginate solution. The egg white powder was dispersed into deionized water (1:10 and 1:15, *w/v*) by a magnetic stirrer for 15 min, filtering out the insoluble particles, then the pH of the solution was adjusted to 10 by adding NaOH pellets. Glycerol (30 wt.% of egg white powder) was added into the prepared protein solution and then we stirred the solution for 15 min at 80 °C to increase the strength and flexibility of the coating as well as oxygen permeability. We added egg yolk powder (15 wt.% of egg white powder) into the solution and stirred for 5 min at the same temperature to alleviate the susceptibility to moisture. Subsequently, the mixed powder of ε-polylysine (0.05%, *w/v*), chitosan (0.1%, *w/v*), and tea polyphenol (0.025%, *w/v*) predispersed in 5 mL deionized water was incorporated, then stirred for another 5 min. Lastly, preprepared clear alginate solution was dispersed in the solution for about 5 min. The mixture was cooled at room temperature for 30 min. To produce the coating film, the solution was poured onto the plate and air-dried at RT for 2~3 days. Cross-linking was performed with 2% (*w/v*) calcium chloride solution, that is, the fillets were first immersed in the coating solution and then immersed in the calcium chloride solution (Figure 1). The concentration of different antimicrobial agents was selected based on the results of minimum inhibitory concentration and initial antimicrobial experiments, and detailed procedures are described in the Supplementary Materials.

### 2.3. Sample Treatment and Storage

Fresh live hybrid sturgeons (*Acipenser baeri♀* × *Acipenser schrenckii♂*) with an average weight and length of 4261 ± 169.3 g and 56.7 ± 7.2 cm were obtained from a local market (Beijing, China), and processed and filleted (weighing 153.6 ± 8.74 g each, *n* = 120) according to the method of Tan et al. [22]. All practices performed in sturgeon transportation and stunning were in strict accordance with the instruction of the World Organisation for Animal Health [23]. The sturgeons were stunned by tapping on the head, scaled, gutted, and washed with cold sterile water immediately. After that, the fillets were drained on sterile gauze for 15 min, then they were manually filleted into cubes (4 × 3 × 2.5 cm^3^) and randomly divided into four treatment groups (*n* = 30 in each group): (1) Control (uncoated); (2) H (coated with 2% sodium alginate combined with antibacterial agents); (3) HP-10 (coated with 2% sodium alginate combined with antibacterial agents + protein solution, egg white powder was dispersed into deionized water 1:10 (*w/v*)); (4) HP-15 (coated with 2% sodium alginate combined with antibacterial agents + protein solution, egg white powder was dispersed into deionized water 1:15 (*w/v*)). Fillets were immersed in the coating solution (fillet/solution ratio of 1:4 (*w/v*)) for 10 min at 4 °C, and the control group CK was immersed in distilled water (Figure 1). After drying for 30 min on presterilized gauze in an ultra-clean workbench, the samples were individually placed in CLEANWRAP fresh-keeping bags and stored at 4 ± 1 °C. Three fillets from each treatment group were randomly selected for analysis on days 0, 2, 4, 6, 8, 12, and 16. 

### 2.4. Rheological Properties of the Coating Solution 

Rheological properties were tested using a controlled shear rheometer (DHR, TA Instruments, New Castle, DE, USA) equipped with a parallel plate (20 mm diameter, 1 mm gap). Coating solutions were taken and placed on a Peltier plate, and covered with a thin layer of silicone oil to prevent evaporation. Then, coating solutions were stabilized at 25 °C for 30 s before measurement, holding at this temperature for 20 min. The relationships between stress and shear rate, and viscosity and shear rate were assessed by steady shear experiments at 25 °C in the shear rate range of 0.1–10 s^−1^. All measurements were performed in triplicate. 

### 2.5. Antimicrobial Evaluation of Coating Solution

Colonies were inoculated into Tryptone Soy Broth (TSB) medium at 30 °C, 160 rpm for 6−8 h; then, 2 mL of culture was centrifuged at 4 °C for 2 min (8000× *g*) and washed three times with sterile water. The cell suspension was adjusted to 10^7^–10^8^ CFU/mL and 0.2 mL was spread on TSB agar plates. Then, 50 μL of coating solution was added to the plates, which were moved to the biochemical incubator (MQD-S3R, Shanghai Minquan Instrument Co., Ltd., Shanghai, China) after standing for 5 min. After incubating at 30 °C for 24 h, the antimicrobial activity was finally evaluated via observing the inhibition zone around the coatings.

### 2.6. Scanning Electron Microscopy (SEM)

Films were sputter-coated with gold and fixed on the sample stage. The microstructures of the surface and the cross-sections of the films were observed using SEM (Hitachi S-4800, Tokyo, Japan) at 4500× magnifications with an accelerating voltage of 7.0 kV. 

### 2.7. X-ray Diffraction (XRD) 

The XRD patterns were determined using a Bruker ray diffractometer (Bruker AXS Inc., Karlsruhe, Germany) according to Rong et al. [24]. The films were tightly packed into an aluminum sample holder and tested in the 2θ range of 8–80° with a scan rate of 4°/min, using Cu Kα radiation (λ = 0.154 nm) operated at a voltage of 40 kV, and a current of 30 mA. 

### 2.8. Film Physical Properties 

#### 2.8.1. Film Thickness

The thickness of films was determined by a micrometer (Chengdu Chengliang Co., Ltd., Chengdu, China). The average value of the film was obtained from 6 measurements at different locations. 

#### 2.8.2. Swelling Capacity

The films were cut into pieces (40 mm × 10 mm), and dried at 105 °C to obtain the initial dry matter weight (m_1_). Then, they were immersed in 30 mL of deionized water for 24 h at 25 °C, taken out of the water, wiped off the water on the surface, and then we measured the weight (m_2_). The swelling capacity (SC) was calculated with the following equation:

SC (%) = (m_2_ − m_1_)/m_1_ × 100 (1)

#### 2.8.3. Water Solubility

The film solubility in water was determined according to the modified method proposed by Li et al. [25]. Three pieces of each film (40 mm × 10 mm) were dried in an oven at 105 °C to a constant weight (m_d_). After immersing in 30 mL of deionized water for 24 h, the undissolved films were taken out and dried to a constant weight (m_f_). The water solubility (S) of film was calculated with the following equation:

S (%) = (m_d_ − m_f_)/m_d_ × 100 (2)

#### 2.8.4. Light Transmittance

The film sample was cut into pieces (45 mm × 10 mm), and placed vertically into the inner wall of the cuvette. Then, the cuvette was placed into the UV spectrophotometer (Cary 50, Shanghai, China), and we measured the light transmittance of the film at the full visible light spectrum (from 400 nm to 800 nm).

#### 2.8.5. Water Vapor Transmission Rate (WVTR)

The WVTR of the films was measured according to the modified method proposed by Li et al. [25]. Briefly, the film was tightly placed over the mouth of a 50 mL centrifuge tube filled with 20 g anhydrous silica gels. We placed the tube in a desiccator containing saturated NaCl solution at 25 °C (relative humidity was 75%). The weight of the silica gel in the centrifuge tube was measured every 3 h until the weight change was less than 0.001 g. The WVTR was calculated as follows:

WVTR[g/(h·m^2^·kPa)] = M / (t × A × ∆*p*) (3)

where M is the added weight of the silica gel (g), t is the time (h) for the weight gain of the silica gel, A is the area of the film exposed to the moisture transfer (m^2^), and ΔP is the water vapor pressure difference between two sides of film sample (kPa). 

### 2.9. Microbiological Analysis 

Microorganisms in each sample were analyzed according to Gui et al. [1,26], including total viable counts (TVC), psychrophilic bacteria, *Pseudomonas*, *Aeromonas*, H_2_S-producing bacteria, lactic acid bacteria, Enterobacteriaceae, and *Vibrio*. Briefly, minced sturgeon (10 g) was transferred aseptically to 90 mL of sterile physiological saline (0.85%, *w/v*) and homogenized for 60 s. For microbial enumeration, 0.1 mL samples of serial dilutions (1:10, 0.85% physiological saline) of fish homogenates were spread on the surface of dry media. All experiments were performed 6 times in parallel, and the microbial counts were expressed as log CFU/g. 

### 2.10. Sensory Evaluation 

The appearance, texture, and odor of sensory properties were evaluated as described by Tan et al. [22]. The scores of 8.0–9.0, 6.0–7.9, 4.0–5.9, and 1.0–3.9 indicate good quality, acceptable quality, spoiled quality, and completely spoiled, respectively.

### 2.11. Determination of pH and TVB-N

The pH of fillets was detected using a digital pH meter (Mettler Toledo FE20/EL20, Shanghai, China) according to the method of Gui et al. [1]. The TVB-N was extracted and determined according to semi-micro steam distillation method in the China National Food Safety Standard methods (Determination of Total Volatile Basic Nitrogen in Food; GB/T 5009.228–2016). The homogenized samples (10 g) were measured by distillation using a Kjeldahl Apparatus (KDY-9820, Beijing, China). The value was expressed as mg of TVB-N per 100 g of flesh. 

### 2.12. Determination of K-Value

ATP-related compounds were extracted and analyzed according to the method of Huang et al. [27]. Briefly, 5 g of sample was homogenized with 10 mL 0.6 M cold perchloric acid (PCA) solution for 1 min, and centrifuged at 10,000 g for 10 min. it was then extracted twice and combined with supernatant, then the pH was adjusted to 6.50 ± 0.05 with NaOH solution. This was followed by centrifuging at 10,000 g for 15 min, and the precipitate was washed with 0.6 M neutralized PCA (pH 6.5). The supernatant was combined and made up to 50 mL using neutralized PCA and stored at −20 °C for further analysis. A high-performance liquid chromatograph (Shimadzu LC-20 series, Kyoto, Japan) equipped with SPD-20A detector and a Shim-pack VP-ODS column (2.0 mm ID × 250 mm × 5 μm) was used to determine the content. The sample was injected with 20 μL and eluted with 0.05 M phosphate buffer (pH 6.8) at 0.2 mL/min, and the absorbance was detected at 254 nm. The adenosine triphosphate (ATP), adenosine diphosphate (ADP), adenosine monophosphate (AMP), inosine monophosphate (IMP), hypoxanthine riboside (HxR), and hypoxanthine (Hx) were determined and calculated based on the retention time and peak area compared with standard solutions, and K-value was defined by the following equation:

K-value (%) = [(HxR + Hx) / (ATP + ADP + AMP + IMP + HxR + Hx)] × 100(4)

### 2.13. Thiobarbituric Acid (TBA) and SDS-PAGE Analysis 

The TBA was used to determine lipid peroxidation in samples, which was detected using a malondialdehyde (MDA) Assay Kit (Nanjing Jiancheng Bioengineering Institute, Nanjing, Jiangsu, China), and the results were expressed in mg MDA/kg flesh. The SDS-PAGE was carried out as described in our previous study [22].

### 2.14. Statistical Analyses

The statistical differences among treatments were tested performing one-way analysis of variance (ANOVA) with Duncan’s test using software SPSS version 21.0 (SPSS Inc., Chicago, IL, USA). Values of *p* < 0.05 were considered as significantly different.

## 3. Results and Discussion

### 3.1. Properties of the Coating Solution

**Figure 2:**
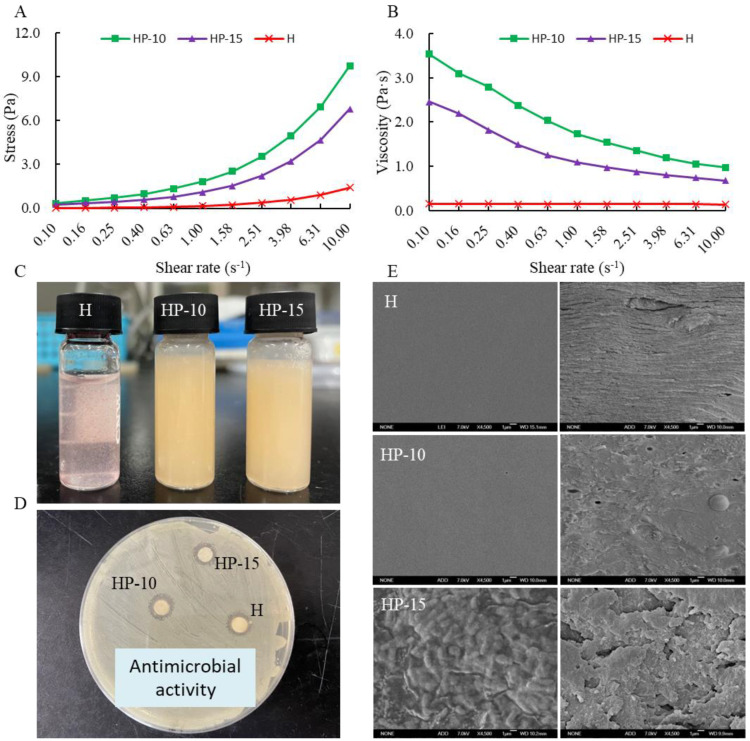
The characterization of coating solution and film. (**A**) The relationship between stress and shear rate, (**B**) the relationship between viscosity and shear rate, (**C**) the different coating solutions, (**D**) the antimicrobial activity of coating solutions, and (**E**) the micrograph (SEM) of the surface and the cross-section of films (H: 2% sodium alginate + antibacterial agents; HP-10: 2% sodium alginate + antibacterial agents + 1:10 protein solution; HP-15: 2% sodium alginate + antibacterial agents + 1:15 protein solution).

The relationship between stress and shear rate is shown in Figure 2A. It shows that the stress of coating solution increased with the increase of shear rate, and the addition of egg white powder can significantly increase the shear stress of the coating solution. Figure 2B shows that the viscosity of coating solution decreased with the increase of shear rate, a shear-thinning behavior, which was defined as a pseudoplastic fluid [28]. The observed flow properties are similar to many starch–polysaccharide and protein–polysaccharide systems [24,29]. Compared with H and HP-15 treatments, the addition of egg white powder can increase the viscosity of the coating solution. In addition, the viscosity of HP-15 coating solution was increased with the addition of more egg white powder. The positive effect of egg protein on the viscosity of the mixture system is due to the interaction between protein and polysaccharide. The appearances of different coating solutions are shown in Figure 2C. 

Figure 2D shows the antimicrobial activities of the different coating solutions against *Aeromonas sobria*. *A. sobria* is a specific spoilage organism (SSO) in refrigerated (4 °C) sturgeon fillets, which is considered to be the main contributor to sturgeon spoilage. Results indicated that each coating solution showed a good inhibitory effect on *A. sobria*, and the addition of egg protein did not affect its antibacterial activity. This suggests that the protein–polysaccharide system might effectively inhibit the spoilage microorganisms in sturgeon fillets. 

### 3.2. Characterization of the Films

The surface and cross-section morphology of the films were observed by SEM, as shown in Figure 2E. The surface morphologies of the H and HP-10 films were smooth and uniform, and the cross-section was also relatively compact. The addition of 1:10 protein solution (*w/v*) improved the compactness and flexibility of the film. However, rough and uneven appearance appeared on the surface of the HP-15 film, and the film compactness was decreased. This indicates that the compactness and uniformity of the film are related to the concentration of egg white powder, which may be due to its random organization of denatured proteins. In addition, the formation of aggregates is also related to the dispersion of antimicrobial agents in the matrix [30]. 

**Figure 3:**
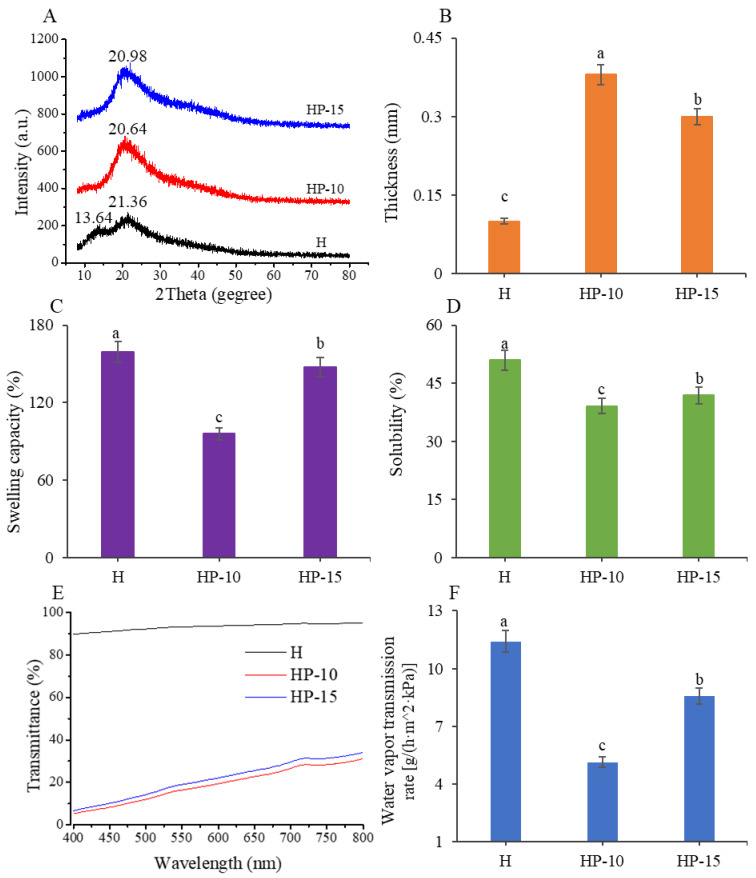
Analysis of physical properties of the films. (**A**) The XRD patterns, (**B**) thickness, (**C**) swelling capacity, (**D**) water solubility, (**E**) light transmittance, and (**F**) water vapor transmission rate of the films (CK: control; H: 2% sodium alginate + antibacterial agents; HP-10: 2% sodium alginate + antibacterial agents + 1:10 protein solution; HP-15: 2% sodium alginate + antibacterial agents + 1:15 protein solution). Different lowercase letters indicated significant differences at *p* < 0.05.

Next, we investigated the crystal structure and compatibility of films using XRD analysis. The XRD patterns can reflect the crystallite size and crystal form through the diffraction peaks [31]. Figure 3A shows that the H film exhibited a strong characteristic peak at 2θ = 21.36° and a lower diffraction peak at 2θ = 13.90°, which was consistent with the characteristic peaks of pure sodium alginate [32], which indicated that the main component of H film was sodium alginate. However, the HP-15 and HP-10 films showed a special characteristic peak only at 2θ = 20.98° and 2θ = 20.64°, respectively. The results indicate that egg protein has good compatibility with sodium alginate. In addition, compared with H film, the intensity of the protein-based film characteristic diffraction peak increased with the addition of more egg white powder. However, the increase in crystallinity facilitates the reduction of solubility of a polymer, which is beneficial for its application in fish meat [31]. 

Furthermore, the thickness, swelling capacity, water solubility, and light transmittance of films were investigated. As shown in Figure 3B, the thickness of HP-15 and HP-10 films were significantly higher than that of H film (*p* < 0.05). Compared with H film, the thickness values for HP-15 and HP-10 films were increased from 0.1 mm to 0.3 and 0.38 mm, respectively. The increase in thickness might be related to the higher solid content per unit surface [25]. From the data on swelling capacity and water solubility (Figure 3C,D), the HP-10 film has the lowest value, indicating that its food preservation effect is better. The previous studies indicated that the solubility of the film is related to its solid content and crystallinity [31]; this is why the addition of 1:10 egg white powder (*w/v*) reduced the water solubility of the composite film. 

To investigate the light barrier properties of the films, experiments for their light transmittance were carried out at a wavelength of 400 nm to 800 nm. As shown in Figure 3E, the light transmittances of protein-based films were significantly decreased, which was mainly attributed to the aromatic amino acids in protein [25]. This is because these amino acids can affect the mechanical properties of the films. Therefore, our results suggested that the HP-15 and HP-10 films were effective in preventing light, which can be used to reduce light-related quality losses. 

The WVTR is another important parameter of films used in food preservation, which can reflect the ability to prevent moisture transfer. As shown in Figure 3F, as the addition of egg white powder increased, the WVTR decreased significantly (*p* < 0.05). The HP-10 film had the lowest water vapor transmission rate of 5.13 g/(h·m^2^·kPa). The excellent moisture barrier capacity of the HP-10 film is related to its compactness and uniformity. This might be because the addition of egg white powder improved the internal structure of films, thereby preventing the passage of water molecules [25]. 

### 3.3. Assessment of Microbial Spoilage in Sturgeon Fillets 

**Figure 4:**
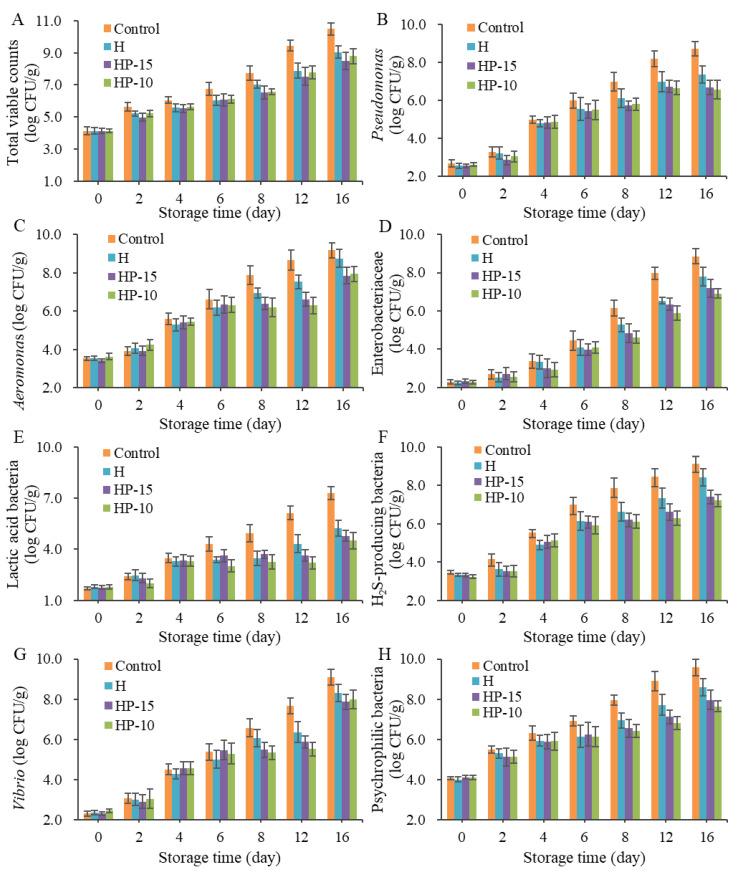
Changes in microbes of sturgeon fillets during refrigerated storage (4 °C). (**A**) Total viable counts, (**B**) number of *Pseudomonas*, (**C**) number of *Aeromonas*, (**D**) number of Enterobacteriaceae, (**E**) number of Lactic acid bacteria, (**F**) number of H_2_S-producing bacteria, (**G**) number of *Vibrio*, (**H**) number of Psychrophilic bacteria (CK: control; H: 2% sodium alginate + antibacterial agents; HP-10: 2% sodium alginate + antibacterial agents + 1:10 protein solution; HP-15: 2% sodium alginate + antibacterial agents + 1:15 protein solution).

To investigate the microbial spoilage of sturgeon fillets after being treated with composite coatings, microbiological analysis was performed. Figure 4 shows the bacterial changes in sturgeon fillets during refrigerated storage (4 °C). The initial TVC for all samples was approximately 4.0 log CFU/g, which means the fillets were of good quality; then the number was increased significantly with storage time (*p* < 0.05). A previous study indicated that the acceptable upper limit of TVC for consumers was 7 log CFU/g [1]. Therefore, the shelf life of control and coating-solution-treated fillets was fewer than 8 and 12 days, respectively (Figure 4A). The composite coatings showed a strong effect in inhibiting the growth of microorganisms. 

*Pseudomonas* and *Aeromonas* have been demonstrated to be the main spoilage bacteria in many freshwater fish. As shown in Figure 4B, the *Pseudomonas* counts were increased dramatically (*p* < 0.05) in all samples, especially the control group, which reached 7.0 log CFU/g on day 8, and the composite coatings (HP-15 and HP-10) exhibited better antimicrobial activity than H, although H reached 7.0 log CFU/g up to day 12. *Aeromonas* is considered as the specific spoilage bacteria in refrigerated sturgeon fillets; the *Aeromonas* counts in the control group were significantly increased (*p* < 0.05) and exceeded 7.0 log CFU/g on day 8, and at the end of storage, the *Aeromonas* was up to 9.2 log CFU/g. In contrast, the TVC in coating-treated groups HP-15 and HP-10 was lower than 7.0 log CFU/g after 12 days of refrigeration, and were 6.6 and 6.3 log CFU/g, respectively (Figure 4C). The results demonstrated that composite coatings have a strong inhibitory effect on *Aeromonas* in fillets. This inhibition of *Aeromonas* and *Pseudomonas* might be ascribed to their sensitivity to anaerobic environments and loss of competitive advantage during storage [1]. 

The initial Enterobacteriaceae in sturgeon fillets were very low, and increased significantly after 4 days of refrigeration. At the end of storage, the number of Enterobacteriaceae in the control group reached 8.9 log CFU/g (Figure 4D). This suggested that Enterobacteriaceae was also the dominant microbe in the later stage of fillets, so their spoilage potential should be considered. In this study, the counts of HP-10 and HP-15 were significantly lower than those of the control group. As for lactic acid bacteria (LAB), their counts were lower than those of Gram-negative bacteria (Figure 4E). The counts of LAB in HP-15 and HP-10 groups remained below 5 log CFU/g, although the control group increased significantly to 7.3 log CFU/g on day 16, and the inhibitory effect of HP-10 was better than that of the HP-15 and the H group. The excellent antibacterial activity against LAB of the composite coatings might be a benefit of a reduced-oxygen atmosphere [33]. 

The number of H_2_S-producing bacteria was increased significantly during refrigerated storage, as shown in Figure 4F. The existence of H_2_S-producing bacteria is an important off-odor contributor in spoiled fillets. Zhuang et al. [34] reported that the rapid increase in H_2_S might be attributed to the microbial metabolism of sulfur-containing amino acids, such as *Shewanella*, the major H_2_S-producing bacteria in many fish. The HP-15 and HP-10 also showed a good inhibitory effect on them. 

*Vibrio* is a typical bacteria found in marine fish but not in freshwater fish. In this study, the initial counts of *Vibrio* were around 2 log CFU/g, and showed a significant increase during the storage (Figure 4G). Although the composite coatings also had an inhibitory effect on the growth of *Vibrio*, it was reported to increase sharply on the 16th day of storage. This suggested that *Vibrio* was also a dominant bacterium at the end of refrigeration. 

The changing trend of psychrophilic bacteria in composite-coatings-treated groups was similar to *Pseudomonas*, H_2_S-producing bacteria, and Enterobacteriaceae (Figure 4H). Psychrophiles are considered to be the main spoilage bacteria in many species of fish due to their ability to survive in low temperatures. They have a special cell membrane structure and contain cold-resistant compounds [1]. Overall, composite coatings have been demonstrated to inhibit the growth of microbes in sturgeon fillets, and the discrepancy between HP-15 and HP-10 might be due to the different physical properties of the coatings. 

### 3.4. Changes in Quality and Biochemistry of Sturgeon Fillets

#### 3.4.1. Sensory Evaluation 

**Figure 5:**
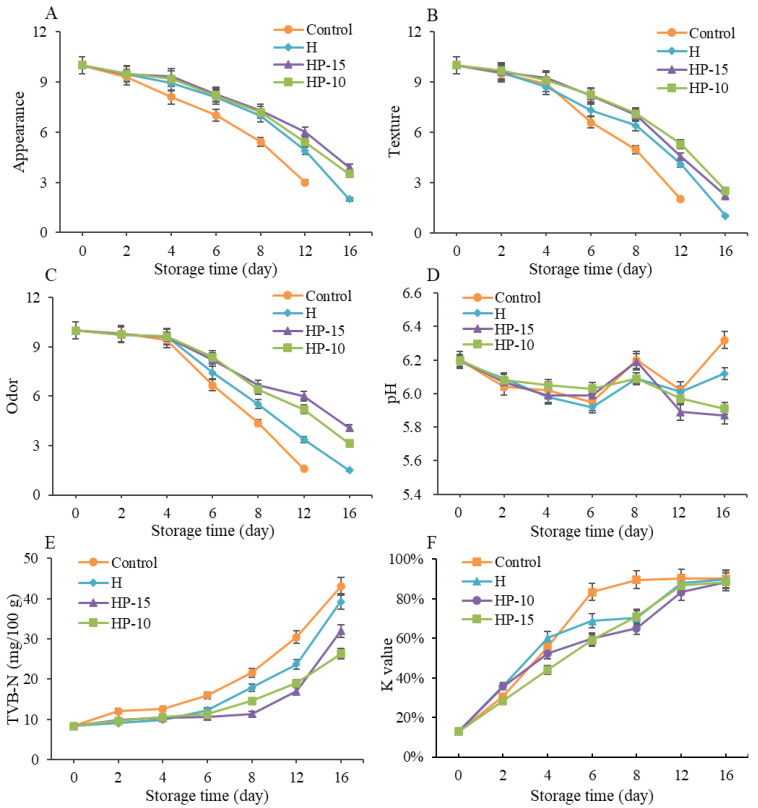
Changes in quality and biochemistry of sturgeon fillets during refrigerated storage. (**A**) Appearance, (**B**) texture, (**C**) odor, (**D**) pH value, (**E**) total volatile basic nitrogen (TVB-N), and (**F**) K-value (CK: control; H: 2% sodium alginate + antibacterial agents; HP-10: 2% sodium alginate + antibacterial agents + 1:10 protein solution; HP-15: 2% sodium alginate + antibacterial agents + 1:15 protein solution).

To further investigate the effect of composite coatings on the quality of refrigerated sturgeon fillets during storage, sensory evaluation was carried out. As shown in Figure 5A–C, the initial sensory quality in all samples was in a state of complete freshness, and the sensory scores decreased gradually with storage time. The sensory quality of the control group was close to unacceptable after 8 days of storage, as the overall score of sensory evaluation below 5 was considered sensory rejection [27], and the sturgeon fillets were completely unacceptable (score below 3) after 12 days of refrigeration, with pungent fishy odors accompanied by plenty of liquid. However, the sensory evaluation of HP-15 and HP-10 was significantly better than the control and H, although, compared to the control, the H group also had better sensory qualities. On day 12 of refrigeration, the sensory scores of HP-15 and HP-10 were still higher than 5, indicating that the sensory shelf life of the coated sturgeon fillets was more than 12 days. The results indicated that the composite coatings could effectively maintain the quality of sturgeon fillets during refrigerated storage. This may be due to the antimicrobial, antioxidant, light, and gas barrier effects of the coatings [31]. 

#### 3.4.2. Changes in pH, TVB-N, and K-Value

The pH changes of refrigerated sturgeon fillets are shown in Figure 5D. The initial pH in all samples was close to 6.2 and a gradual decrease of the pH occurred within the first 6 days, then fluctuations were observed during the following storage. The pattern of changes in control was consistent with previous studies [1,22]. Many researchers point out that the initial decrease of pH may result from the lactic acid and pyrophosphate accumulation, while the later increase in pH is due to the accumulation of protein degradation metabolites (e.g., ammonia and biogenic amines) that are produced by endogenous microorganisms [4,35]. Compared with control, lower pH values were presented in H, HP-15, and HP-10 treatments during later storage, and the HP-15 and HP-10 had a lower pH than H. This result indicated that the composite coatings could reduce the generation of alkaline compounds (such as biogenic amines and ammonia) by inhibiting the growth and metabolism of microorganisms, and the addition of egg white powder can enhance this effect. This was also demonstrated in the previous studies [22].

The content of TVB-N is an important direct indicator to assess fish spoilage, which is related to the degradation of protein or nonprotein nitrogenous compounds. As shown in Figure 5E, the initial TVB-N values in all samples were very low and increased continually with storage time. In the control group, the TVB-N content of sturgeon fillets was significantly increased during storage (*p* < 0.05), exceeding the unacceptable threshold (21.56 mg/100 g) on the 8th day and reaching 43.05 mg/100 g on the 16th day. Compared with control, the final TVB-N values of H, HP-15, and HP-10 were reduced by 8.8%, 25.7%, and 38.9%, respectively. In addition, the TVB-N values of HP-15 and HP-10 only reached 16.94 and 18.97 mg/100 g on day 12, which was still lower than the acceptable limit of 20 mg/kg for refrigerated fish fillets [36]. This suggested that the alginate antibacterial coating in combination with egg white powder provided a better preservation effect than the alginate antibacterial coating alone. 

The K-value was carried out to evaluate the freshness of refrigerated sturgeon fillets, as shown in Figure 5F. The K-value of all samples was increased gradually with storage time, and the control and treatments reached a leveling-off period after 8 and 12 days of refrigeration, respectively. The coating-solution-treated samples presented significantly lower (*p* < 0.05) K-values than the control during refrigeration. The best treatment to prevent nucleotide degradation during the first 6 days is HP-15; after that it is HP-10. The results further indicated that alginate antibacterial coating in combination with egg white powder was effective in maintaining a better quality of fish. This might be because the coating inhibited the activities of microorganisms, thereby affecting the degradation of IMP and the accumulation of Hx and HxR [37]. A previous study indicated that the degradation of ATP-related compounds was mainly caused by both autolytic and microbial enzymes [4]. According to the evaluation criterion of Özogul et al. [38], the fish quality is considered unacceptable when the K-value is higher than 85%. Therefore, based on K-value, the shelf life of control and coating-treated samples were about 8 and 12 days, respectively. The results were consistent with sensory evaluation and TVB-N analysis. 

#### 3.4.3. Changes in TBA 

**Figure 6:**
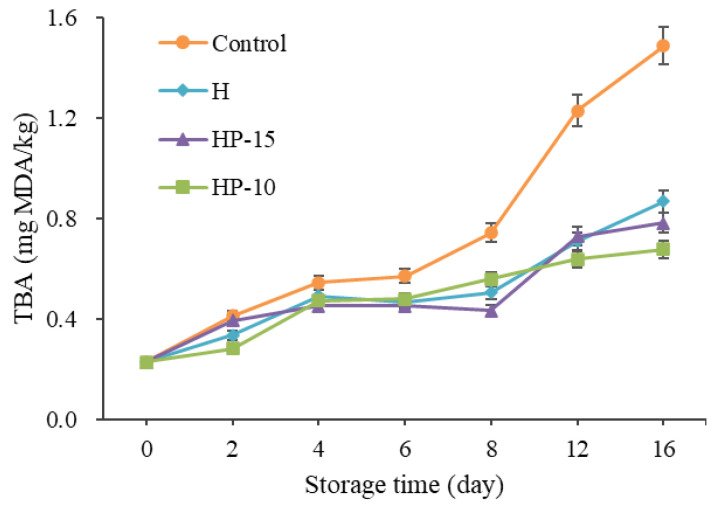
Changes of TBA in sturgeon fillets during refrigerated storage (CK: control; H: 2% sodium alginate + antibacterial agents; HP-10: 2% sodium alginate + antibacterial agents + 1:10 protein solution; HP-15: 2% sodium alginate + antibacterial agents + 1:15 protein solution).

The change of TBA value corresponds to the malondialdehyde (MDA) value of peroxide decomposition in fillets. It is widely used to evaluate lipid oxidation [39]. In this study, the initial TBA values in all samples were 0.23 mg MDA/kg, and increased gradually to 1.49, 0.87, 0.78, and 0.68 mg MDA/kg after being stored for 16 days in control, H, HP-15, and HP-10 samples, respectively (Figure 6). The TBA values of the coating-treated samples were significantly lower than those of the control group (*p* < 0.05), and HP-10 worked better. This result indicated that the composite coatings had significant antioxidant activity, and the delay of the composite coatings on lipid oxidation in sturgeon fillets may be due to its barrier properties [4]. According to Cai et al. [40], the factors leading to lipid oxidation are mainly oxygen and pro-oxidants in fish flesh; they can react with muscle components. Behbahani et al. [21] demonstrated that edible coating may delay the oxidation probably due to the antioxidant activity of the phenolic compounds and the ability of the edible coating to minimize the contact with oxygen and light. Accordingly, this suggested that in fish preservation, the HP-10 coating acted as an antioxidant by effectively shielding oxygen and light. 

#### 3.4.4. SDS-PAGE Analysis 

**Figure 7:**
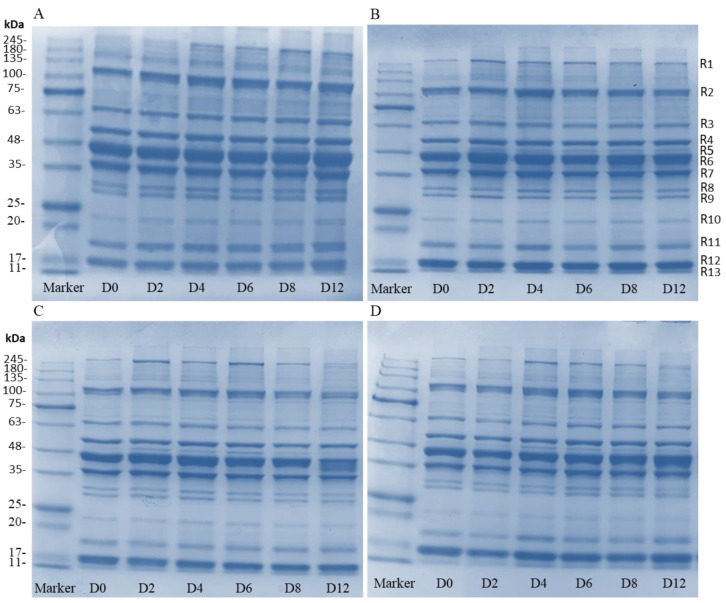
The SDS-PAGE analysis of control samples (**A**), H samples (**B**), HP-15 samples (**C**), and HP-10 samples (**D**) (CK: control; H: 2% sodium alginate + antibacterial agents; HP-10: 2% sodium alginate + antibacterial agents + 1:10 protein solution; HP-15: 2% sodium alginate + antibacterial agents + 1:15 protein solution). D0 to D12 represent storage on days 0 to 12.

The variations of protein in sturgeon fillets were analyzed, and are shown in Figure 6. All groups possess 13 clear bands (R1–R13) and there were some differences between the control group and the coating-treated groups. In the control group, the band intensity at around 120, 70, 23, 18.5, and 17 kDa (R2, R3, R10, R11, and R12) increased, and was accompanied by bands around 260 and 50 kDa (R1 and R5) (Figure 7A). However, bands of protein in the coating treatments did not change significantly during storage, although there were slight differences in protein concentrations of different molecular weights (Figure 7B–D). The composite coating treatments decreased the concentration of low-molecular-weight proteins when compared with the control and H groups. This change of band intensity indicated that the coatings reduced protein degradation by growth inhibition of microorganisms, which is consistent with the result of Li et al. [41]. According to the previous studies, the bands of 15–20 kDa might be associated with the spoilage bacteria *Aeromonas*, and the *Pseudomonas* and *Shewanella* contributed to the bands of 25–35 kDa [42,43]. The bands of 36 kDa (R6) and 45 kDa (R7) might be related to the degradation of myofibrillar proteins by microbial activity [42]. This study further confirmed that bacterial activities are the main factors responsible for protein degradation in fish fillets.

## 4. Conclusions

This study reports an edible alginate/protein-based coating, incorporated with chitosan, ε-polylysine, and tea polyphenol. The composite coatings exhibited a more stable structure and better light, gas, and water barrier properties. At the same time, the addition of egg white powder increased the formability, stability, and thickness of the coatings or films, and decreased the swelling capacity, water solubility, transmittance, and water vapor transmission rate. The results of physicochemical and sensory quality of fillets indicate that the composite coatings can successfully maintain sensory quality and reduce the degree of chemical spoilage. This is due to the composite coatings that delayed the lipid oxidation and inhibited the growth of microorganisms (including *Pseudomonas*, *Aeromonas*, Enterobacteriaceae, H_2_S-producing bacteria, LAB, *Vibrio*, and psychrophilic bacteria). Thus, they reduced the production of TVB-N and nucleotide breakdown as well as protein degradation in fillets. Therefore, the composite coatings can provide an attractive alternative for freshness preservation of sturgeon fillets or other meats with high fat content.

## Data Availability

Data is contained within the article or Supplementary Materials.

## Supplementary Materials

The following are available online at https://www.mdpi.com/article/10.3390/foods11213380/s1, Figure S1: Inhibitory effect of different concentrations of preservatives on specific spoilage organisms *Aeromonas sobria* LT-101.
