# Supplementary material for: Development of a Multifunctional Edible Coating and Its Preservation Effect on Sturgeon (Acipenser baeri♀ × Acipenser schrenckii♂) Fillets during Refrigerated Storage at 4 °C
Source: Foods. 2022 Oct 27;11(21):3380. doi: 10.3390/foods11213380 (PMC9655360; doi:10.3390/foods11213380)
Supplement: Supplementary file 1 [file foods-11-03380-s001.zip › foods-1975891-supplementary.pdf]

Supplementary file

1. Determination of minimum inhibitory concentration (MIC)

The 64 mg/mL tea polyphenol solution, ε-polylysine solution and chitosan solution were prepared by using sterile water. After filtration with a 0.45 μm sterile filter membrane, 2-fold serial dilution with Tryptone Soy Broth (TSB) to obtain a series of different concentration solutions. The specific spoilage organism *Aeromonas sobria* LT-101 was inoculated in TSB medium and cultured with shaking at 30 °C for 12 h, the final concentration adjusted to 10^6 CFU/mL. Then 180 μL of bacterial solution and 20 μL of tea polyphenols, chitosan, and ε-polylysine solutions of different concentrations were added to 96-well plates, and incubated at 30 °C for 24 h. The absorbance value at 600 nm was measured by using a microplate reader. The lowest concentration that inhibited bacterial growth was taken as the MIC of each antibacterial agent.

2. The effect of different concentrations of antibacterial agents on *A. sobria* LT-101

Taking 40 mL of fish juice into 100 mL conical flasks, added 100 μL of the bacterial solution (10^6 CFU/mL), and then added a certain concentration of tea polyphenols, ε-polylysine, or chitosan. The final concentration of antibacterial agents in fish juice range from 0 to 4 mg/mL. The total viable counts were determined after being shaken and cultured at 140 rpm in an incubator at 30 °C for 24 h. Three replicates were performed for each concentration.

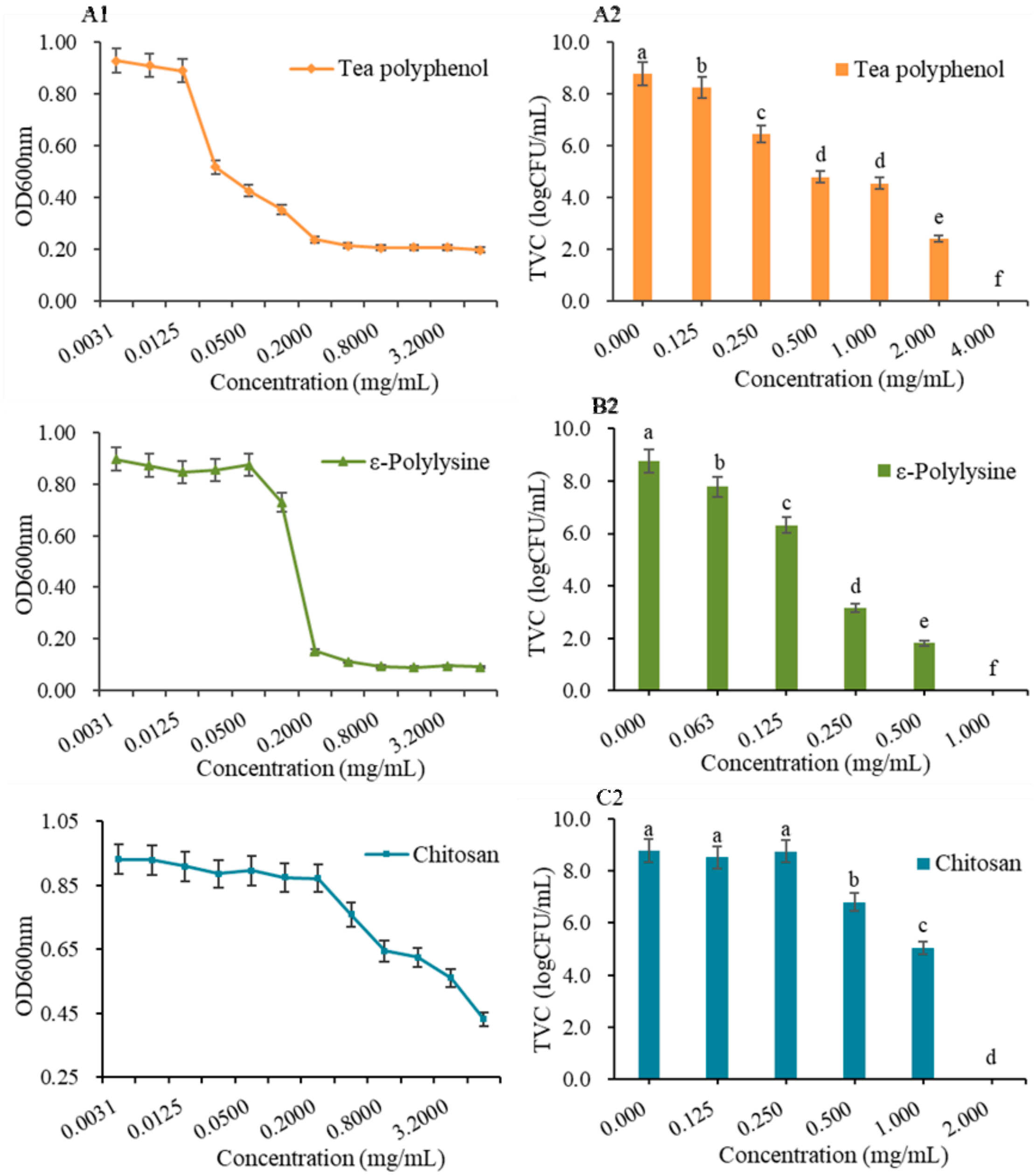

Figure S1. Inhibitory effect of different concentrations of preservatives on specific spoilage organisms *Aeromonas sobria* LT-101.
